# Education on electrical phenomena involved in electroporation-based therapies and treatments: a blended learning approach

**DOI:** 10.1186/s12938-016-0152-7

**Published:** 2016-04-07

**Authors:** Selma Čorović, Samo Mahnič-Kalamiza, Damijan Miklavčič

**Affiliations:** Faculty of Electrical Engineering, University of Ljubljana, Tržaška c. 25, 1000 Ljubljana, Slovenia

**Keywords:** Electric field, Electroporation, E-learning, Item analysis, Electroporation-based technologies and treatments

## Abstract

**Background:**

Electroporation-based applications require multidisciplinary expertise and collaboration of experts with different professional backgrounds in engineering and science. Beginning in 2003, an international scientific workshop and postgraduate course electroporation based technologies and treatments (EBTT) has been organized at the University of Ljubljana to facilitate transfer of knowledge from leading experts to researches, students and newcomers in the field of electroporation. In this paper we present one of the integral parts of EBTT: an e-learning practical work we developed to complement delivery of knowledge via lectures and laboratory work, thus providing a blended learning approach on electrical phenomena involved in electroporation-based therapies and treatments.

**Methods:**

The learning effect was assessed via a pre- and post e-learning examination test composed of 10 multiple choice questions (i.e. items). The e-learning practical work session and both of the e-learning examination tests were carried out after the live EBTT lectures and other laboratory work. Statistical analysis was performed to compare and evaluate the learning effect measured in two groups of students: (1) electrical engineers and (2) natural scientists (i.e. medical doctors, biologists and chemists) undergoing the e-learning practical work in 2011–2014 academic years. Item analysis was performed to assess the difficulty of each item of the examination test.

**Results:**

The results of our study show that the total score on the post examination test significantly improved and the item difficulty in both experimental groups decreased. The natural scientists reached the same level of knowledge (no statistical difference in total post-examination test score) on the post-course test take, as do electrical engineers, although the engineers started with statistically higher total pre-test examination score, as expected.

**Conclusions:**

The main objective of this study was to investigate whether the educational content the e-learning practical work presented to the students with different professional backgrounds enhanced their knowledge acquired via lectures during EBTT. We compared the learning effect assessed in two experimental groups undergoing the e-learning practical work: electrical engineers and natural scientists. The same level of knowledge on the post-course examination was reached in both groups. The results indicate that our e-learning platform supported by blended learning approach provides an effective learning tool for populations with mixed professional backgrounds and thus plays an important role in bridging the gap between scientific domains involved in electroporation-based technologies and treatments.

**Electronic supplementary material:**

The online version of this article (doi:10.1186/s12938-016-0152-7) contains supplementary material, which is available to authorized users.

## Background

Electroporation is a versatile technological platform that allows for controlled administration of different molecules into the biological cells and extraction of cellular components out of cells via application of appropriate electric field [1]. Electroporation is used in various biotechnological applications (i.e. electroporation-based technologies and treatments) including medicine, food processing, and fuel production [2, 3]. The electroporation technology is inherently multidisciplinary and it is based on the knowledge from many disciplines of engineering and science. Namely, the design of electroporation equipment (i.e. electric pulse generators, electrodes and electroporation chambers) requires close collaboration of electrical and computer engineers with professionals from different domains including oncology, chemistry, and process engineering. Similarly, planning of electroporation-based medical applications such as for example electrochemotherapy requires multidisciplinary expertise and close collaboration of experts from medical imaging, numerical modelling of electrical phenomena within cancerous tissues, and in vivo experimental work [4–7].

In order to efficiently make use of electroporation it is of outmost importance for both engineers and natural scientists to know and understand the effects of electric field responsible for electroporation of the treated sample (cells or tissues). Novice learners coming from the natural sciences background should be supported with basic physical concept of electric field distribution in a simplified manner without a detailed scientific theory. On the other hand for electrical engineers it is also of outmost importance to understand basic chemical and biological mechanisms underlying electroporation of cells and tissues. It was therefore necessary to establish a multidisciplinary learning environment to facilitate rapid and efficient transfer of knowledge from one domain to another and to establish a strong scientific collaboration among the scientists. An international scientific workshop and postgraduate course Electroporation based technologies and treatments (EBTT) has been organized biennially from 2003–2009 and annually since 2011 at the University of Ljubljana with the aim of conveying the knowledge from world leading experts in the field of electroporation to students, most of which are Ph.D. students and early stage researchers with different academic backgrounds coming from scientific domains, interested in electroporation [8]. The lectures given during EBTT are complemented by laboratory practical work to support the lectures with the methodologies commonly used in the investigations relevant to the research field. One of the integral parts of laboratory work at EBTT is an e-learning practical work session that we developed to provide an interactive “hands-on” presentation of educational content about important parameters of electric field distribution previously calculated with realistic numerical models [6, 9–11]. The e-learning educational content reinforces the students’ awareness of most of the critical issues introduced to them via lectures, thus providing a blended learning approach. The modular structure of e-learning practical work brings together both experimental and theoretical findings, as well as cutting-edge insights from molecular dynamics simulations, and allows for both continuous upgrades with new content being published within the scientific literature on electroporation based applications and for learning effectiveness evaluation.

We have previously developed and implemented the first version of e-learning practical work on electrical phenomena involved in electrochemotherapy [12]. In this paper we present a more general e-learning practical work platform relevant to learning about electrical phenomena in other electroporation-based therapies and treatments. The main objective of this study was to investigate whether the educational content the e-learning practical work platform presented to the students with different professional backgrounds facilitated learning on electrical phenomena underlying electroporation of cell membrane, single cell, and tissue that are important in better understanding and planning of electroporation-based therapies and treatments. We compared the learning effect measured in two groups of students (i.e. total of 13 electrical engineers and 34 natural scientists) undergoing the e-learning practical work in 2011–2014 academic years. The learning effect was assessed via a pre- and post e-learning examination test composed of ten multiple choice questions (i.e. items). The results of our study show that the total score on the post examination test significantly improved and the item difficulty in both experimental groups decreased. The natural scientists reached the same level of knowledge (no statistical difference in total post-examination test score) on the post-course test take, as do electrical engineers, although the engineers started with statistically higher total pre-test examination score, as expected. The results indicate that our e-learning platform supported by blended learning approach provides an effective learning tool for populations with mixed professional backgrounds and thus plays an important role in bridging the gap between scientific domains involved in electroporation-based technologies and treatments.

## Methods

The e-learning application we describe in this study is the second version of the e-learning practical work platform based on the e-learning application we previously developed and presented to the participants of the EBTT program delivered in academic years 2005–2009. This second version represents a redesigned and expanded e-learning application and was presented to the participants of the EBTT program delivered during the 2011–2014 academic years.

In this section we describe the methodology we used to implement the graphical user interface and to incorporate the educational content that was based on new and recently published theoretical and experimental findings on electroporation of cell membrane, lattice of cells and tissues, including the new findings at the molecular level of cell membrane electroporation. The presentation of content is based on scientific visualization of previously developed theoretical models of electroporation phenomena on different levels: molecular [13, 14], membrane [15], cell [16], and tissue [9]. Scientific visualization focuses on creating approaches for conveying abstract scientific information in intuitive ways [17]. The purpose of applying scientific visualization in our case is to graphically illustrate scientific data, thus facilitating the transfer of complex knowledge to target users not knowledgeable in one or several domains which electroporation is spanning. This for example enables scientists with predominantly biomedical background to more easily gain insight into the processes that govern their experiments and therapies by employing the knowledge from electrical engineering helping them develop a critical outlook on their experimental setups, treatments, and data obtained.

### Ethics statement

The informed consent to participate in the study was obtained from all participants. Before the start of the e-learning practical work all the participants provided their agreement on the use of the results of pedagogical efficiency of the e-learning content on electroporation and usability study of the e-learning practical work for research purposes. Current research practice and legislation in Slovenia do not require an ethical approval to conduct such a study [18].

### The software implementation of the e-learning practical work

The e-learning website is hosted on a semi-open platform. A Microsoft^®^ Windows operating system hosts an Apache™ HTTP Server (a web server) service with PHP support installed and a free edition of Oracle^®^ MySQL service provides for the database backend, which is also commonly known as a WAMP (Windows-Apache-MySQL-PHP) technology stack. The code is mainly written in and adheres to standards of HTML version 4.0, styling of the website is implemented entirely through use of custom style sheets (CSS), and the dynamics of displaying and hiding content interactively by the user are implemented by use of JavaScript, the use of the latter enhanced by limited implementation of the jQuery library. The PHP scripting language provides additional support in cross-site navigation and rewrites addresses dynamically to render them user-readable and understandable, i.e. the user can discern her or his location on the site easily by verifying the current URL (Uniform Resource Locator, i.e. the full website address in the address bar). All user interaction requiring the use of a database and site communication with the database are also implemented through use of PHP. The PHP code is written on top of a base library, which was chosen according to personal preferences, and the preferred framework at the time of application development was CodeIgniter. CodeIgniter is an open-source library that provides functions needed to provide basic functionality with a minimal amount of modifications needed, such as handling of POST and GET requests, management of user session data, cookie management, etc. The functions custom-built in PHP perform the following tasks: gathering and storing data collected during the knowledge evaluation, maintaining the user identification throughout her or his exploration of the site, identifying the user upon the second take of the knowledge test and to commence the session with the evaluation questionnaire, instantaneously generating results of the knowledge test and usability questionnaire in terms of statistical analysis and display for the purposes of analysis by the teacher etc.

**Fig. 1:**
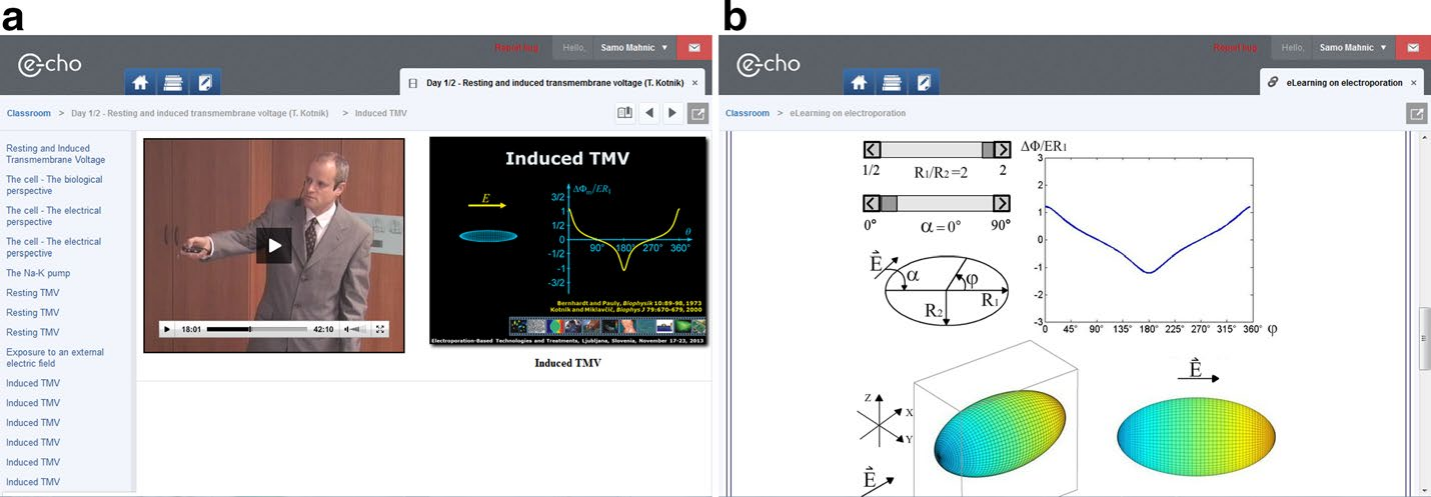
An example of an e-learning session displayed through the E-CHO e-learning environment. An example of an e-learning session displayed through the E-CHO e-learning environment showing: **a** an e-learning ‘E-CHO classroom’ with a video lecture; and **b** the Power point presentation supporting the subject matter given in the video lecture

The e-learning practical work is integrated into an e-learning environment (i.e. E-CHO) developed by the Laboratory of Telecommunications of the University of Ljubljana at the Faculty of Electrical Engineering [19–21]. The E-CHO e-learning environment supports video streaming and facilitates communication among users (i.e. forums, e-mail correspondence and videoconferencing). The e-learning practical work is accessible to students of the EBTT for several months after the course so that the students can review the recorded lectures or re-take the exam to test their knowledge. Figure 1 illustrates the E-CHO environment (Fig. 1a), showing a screenshot of a recorded video lecture given on the subject of induced transmembrane voltage across a single cell. The video lecture is simultaneously accompanied by the Power Point slides providing the textual and graphical explanation of the given lecture (Fig. 1b).

The scientific visualization of the educational content was carried out by means of static and interactive 2D and 3D graphics, images, and animated sequences. The static visualization was designed for the basic level of educational content providing simple and pictorial representation of the electroporation process resulting from the input parameters selected by the instructor to enable the user to learn from the most representative study cases. The interactive scientific visualization enables a simulation of ‘hands-on’ learning enabling intermediate and advanced users to vary different input parameters. The interactive educational content is developed by using 3D studio max (autodesk media and entertainment, Quebec, Canada) and Macromedia Flash 8 interactive animated sequences that were based on previous numerical simulations performed with Matlab (MathWorks, MA, USA), and Comsol Multiphysics (COMSOL AB, Stockholm, Sweden) software packages. To design interactive content based on analytical calculation of electric potential and electric field distribution, we implemented the ApiVizTEP educational application previously developed as a desktop application [22].

### Description of educational content

**Fig. 2:**
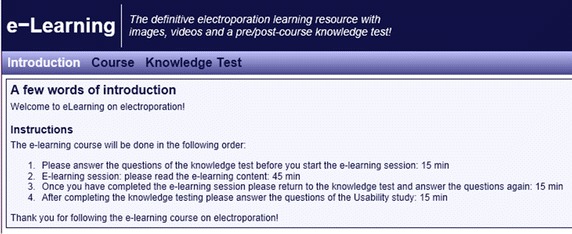
Graphical user interface of the e-learning practical work. Graphical user interface GUI of the second version of the e-learning practical work: introduction *panel*

The graphical user interface of the second version of the e-learning practical work presented in this study is given in Fig. 2.

The default page of the practical work (Introduction panel) introduces the educational content and provides a list of instructions on how to execute the e-learning practical work.

**Fig. 3:**
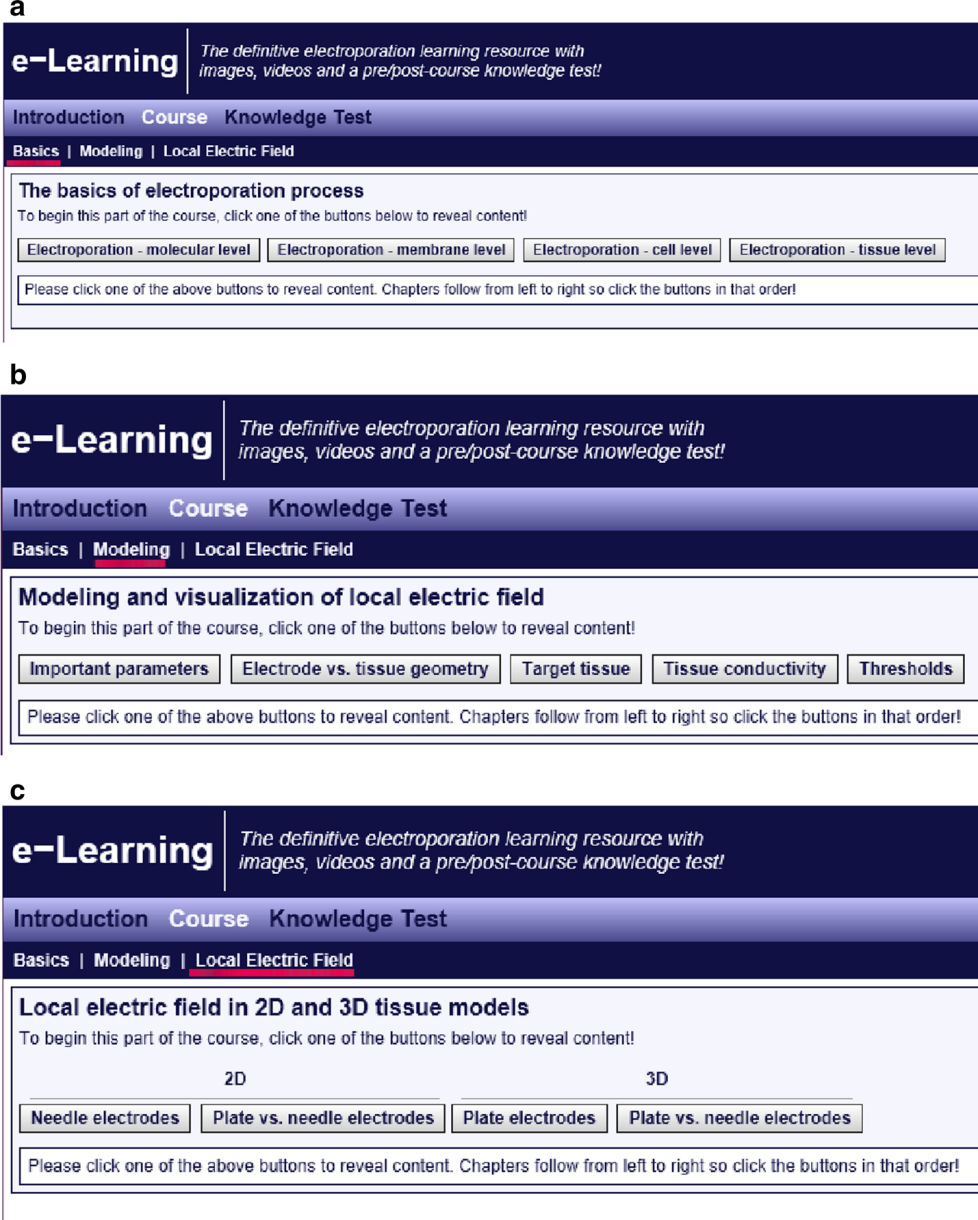
Organisation of the educational content. The main *panel* (i.e. course *panel*) providing the educational content of the e-learning practical work: **a** Basics—basic level of knowledge; **b** Modelling—intermediate level of knowledge, and **c** Local electric field—advanced level of knowledge

The Course panel provides the educational content of the e-learning practical work (Fig. 3), and is composed of three main subpanels that expose the educational content in an increasing order of difficulty: basic (Fig. 3a), intermediate (Fig. 3b), and advanced (Fig. 3c) level of knowledge.

The educational content provided by each of the abovementioned panels is supported with links to relevant publications and by video lectures given during the EBTT school.

**Fig. 4:**
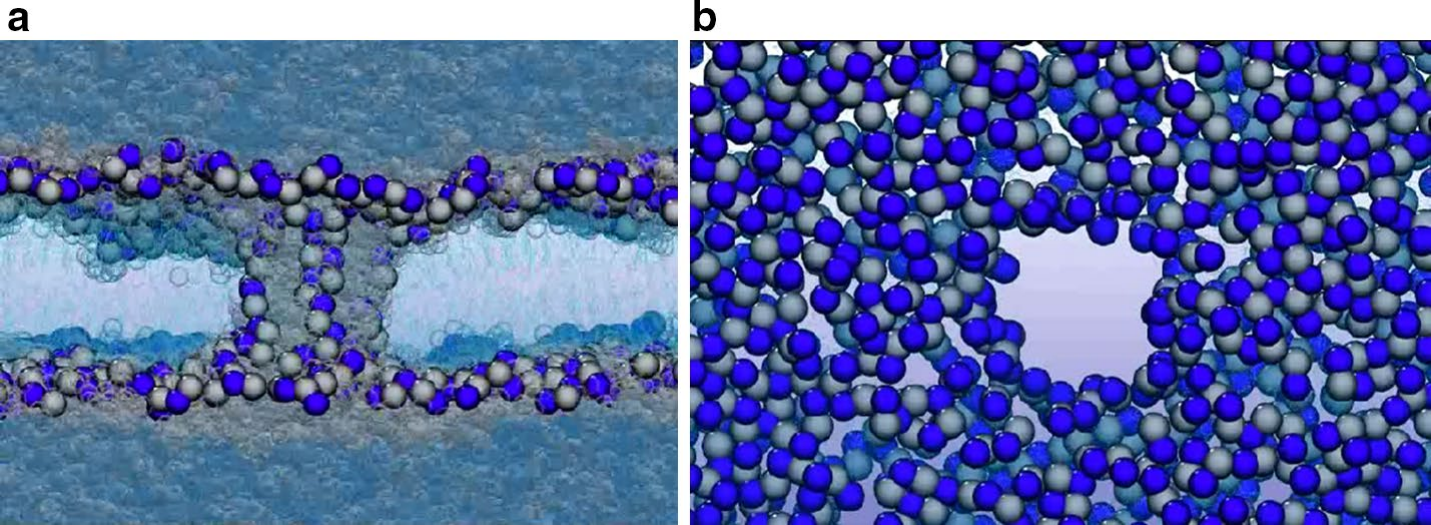
Molecular dynamics simulation of a pore creation throughout the lipid bilayer. Molecular dynamics simulation of a pore creation in the lipid bilayer. *Side view* (**a**) and *top view* (**b**). The water molecules are *light transparent*. The lipid head groups are in *dark blue* and *grey colour* for choline and phosphate, respectively. (Original movies/figures courtesy of Dr. Mounir Tarek, University of Lorraine, Nancy, France). The water molecules are *light blue* and *semi-transparent*. The lipid head groups are in *dark blue* and *grey colour* for choline and phosphate, respectively

The first panel, basics (shown in Fig. 3a), introduces the basic level of knowledge behind the electroporation of cells and tissues at four different levels: molecular, membrane, cell, and tissue level. The educational content of basic knowledge on electroporation phenomena at the level of membrane was extended with the knowledge acquired by recent advances in the field of computational molecular dynamics—currently, the only technique that allows us to analyse the electroporation phenomenon at the atomistic scale and to follow the transport of ions and molecules through electroporated lipid bilayers. We provide scientific visualization of electroporation-induced local perturbation in lipid bilayers in the form of movies showing pore creation in the lipid bilayer (palmitoyl-oleyl-phosphatidyl-choline, POPC) as a result of induced transmembrane voltage by using charge imbalance method. The movies that we incorporated into the educational content are the results of actual computational molecular dynamics simulations. The Fig. 4 shows a pore creation (i.e. water channel) that enables transport of ions and molecules throughout the lipid bilayer, as it was shown in the movies (Fig. 4a side view and Fig. 4b top view).

The educational content on cell level is extended with the basic explanation of resting and induced transmembrane potential. Namely, we point out that under normal conditions (when the electroporation pulses are not applied), a resting transmembrane voltage (RTMV) is present across the cell membrane at all times. The RTMV ranges from between -90 and -40 mV (or less), and depends on cell type and other factors, such as stage of cell differentiation. On the other hand, delivery of sufficiently high electric pulses induces an electric field within the cells or tissues and subsequently creates an induced potential difference (i.e. induced transmembrane voltage or ITMV) across the cell membrane which is superimposed to the RTMV. This results in total transmembrane voltage TTMV, where TTMV = RTMV + ITMV.

**Fig. 5:**
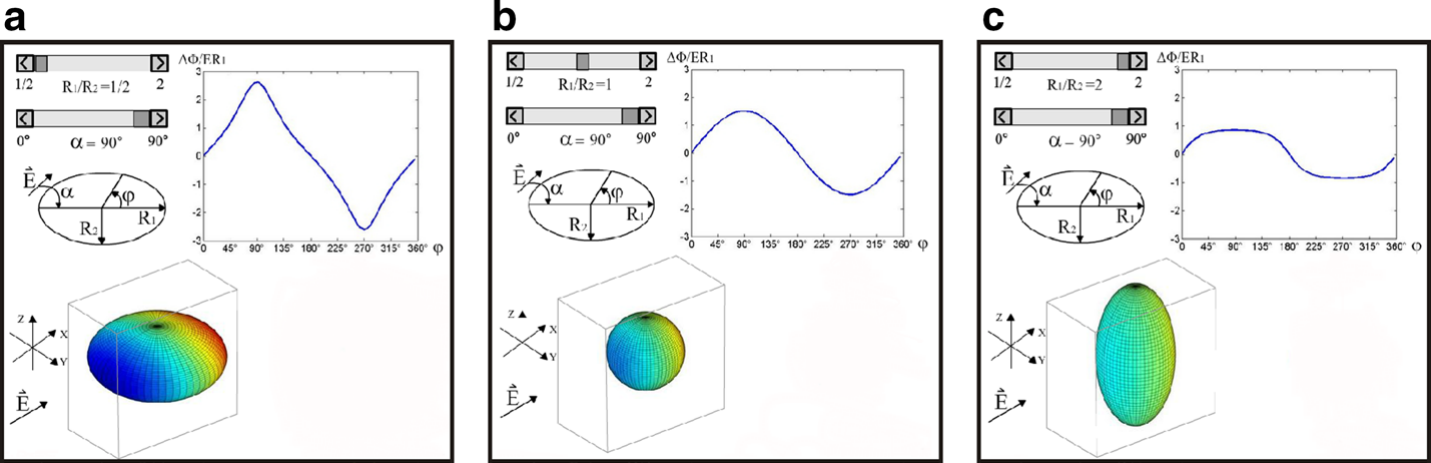
Interactive visualization of induced transmembrane voltage for spheroid-shaped cells. Interactive visualization of induced transmembrane voltage for spheroid-shaped cells with three different geometries: *R*_1_/*R*_2_ = 1/2 (**a**); *R*_1_/*R*_2_ = 1 (**b**), and *R*_1_/*R*_2_ = 2 (**c**). *E* is the electric field and *φ* is the angle between the direction of *E* and the selected point on the cell surface

We proceed with the textual and graphical explanation of analytical solution of Schwann’s equation (for sphere-shaped cells) and provide an interactive application that enables the user to learn how the transmembrane voltage changes if the cell is a prolate or oblate spheroid, and not a perfect sphere (i.e. radius *R*_1_ is not equal to the radius *R*_2_, Fig. 5) enabling the user to compare the changes in induced transmembrane voltage for a spherical cell (*R*_1_ = *R*_2_, Fig. 5), as shown in Fig. 5.

The application also enables the visualization of the transmembrane voltage if a cell is tilted with respect to the electric field, which this is represented by angle *α* in Fig. 5 (*R* is the cell radius, *E* is electric field, and *φ* is an angle between the direction of *E* and the selected point on the cell surface in Fig. 5). The importance of cell orientation with respect to the applied electric field has been demonstrated both in vitro [23] and in vivo [9]. Figure 5 shows transmembrane voltage calculated and visualized for three different ratios of radii: Fig. 5a. *R*_1_/*R*_2_ = 1/2, Fig. 5b *R*_1_/*R*_2_ = 1, and Fig. 5c *R*_1_/*R*_2_ = 2 (*α* is 90° in all cases). By comparing Fig. 5a–c one can observe that the induced transmembrane voltage strongly depends on cell geometry (i.e. the *R*_1_/*R*_2_ ratio). Finally, we point out that the induced membrane voltage induced can be effectively measured experimentally and provided the user with a link to the protocol for non-invasive measurements of transmembrane voltage by using the potentiometric dye di-8-ANEPPS we previously published in journal of visual experiments [16].

**Fig. 6:**
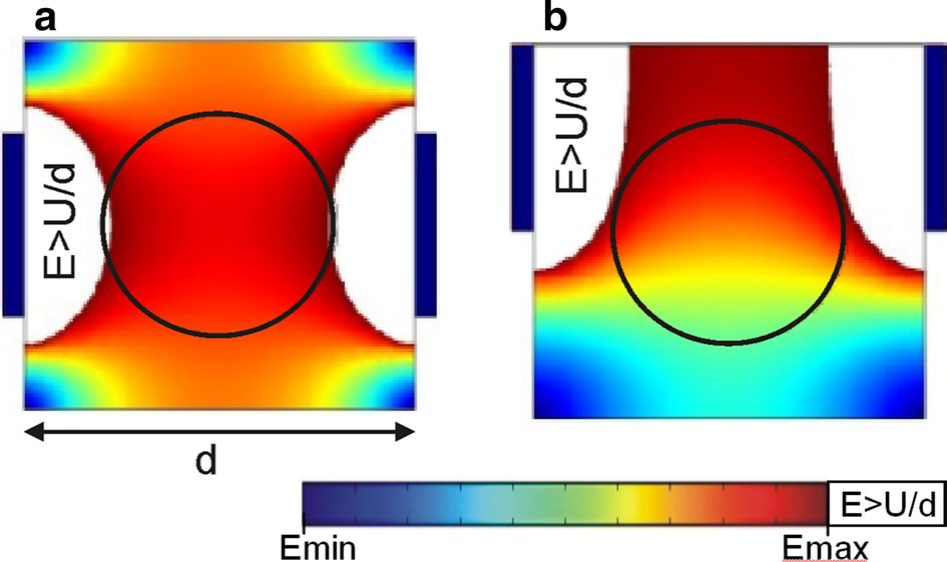
Visualisation of numerically calculated electric field distribution in target tissue in 2D. Static visualisation of numerically calculated electric field distribution in target tissue in 2D (*black circle*) for: (**a**) a symmetrical position and (**b**) a non-symmetrical position of plate electrodes

The second panel, modelling (in Fig. 3b), provides intermediate level of knowledge on modelling and visualization of local electric field distribution in tissues. We introduced the basic concepts in state-of-the-art modelling (i.e. numerical calculations with finite elements method) of the electric field distribution in tissues. This panel provides further explanation on the important parameters that have direct effects on electric field distribution within tissues, which is necessary for better understanding of the educational content that follows in the subsequent panel providing advanced level of knowledge (Fig. 3c). The definition of the important parameters (such as electrode geometry, distance between electrodes, electrode position/orientation with respect to the target tissue, tissue geometry and conductivity, electrode-tissue contact surface, and voltage applied to the electrodes) and their effects on electric field distribution in tissue is given by way of simple pictorial and graphical examples in 2D to motivate the users in learning. For example, Fig. 6 shows a comparison of electric field distribution in target tissue (black circle) and its surrounding tissue for two different electrode positions with respect to the target tissue.

Electric field distribution is given for symmetrical and non-symmetrical electrode positioning, in Fig. 6a, b, respectively. Intensities of the electric field are coded with a colour map ranging from blue (the lowest value) to red (the highest value). The white region in Fig. 6 denotes the tissue area exposed to the electric field higher than the voltage to distance ratio (*U*/*d*)—the value corresponding to the uniform electric field distribution in an ideal configuration of two infinite parallel plate electrodes. The symmetrical position of the electrodes with respect to the target tissue results in a symmetrical electric field distribution, having higher values in the central part of the target tissue (for example tumour) compared to the marginal regions of the circle. On the other hand, if the electrodes are positioned in a non-symmetrical manner, as shown in Fig. 6b, the uppermost area (close to the electrodes) of the circle is subjected to a higher intensity of the electric field, as compared to the lower part of the circle.

**Fig. 7:**
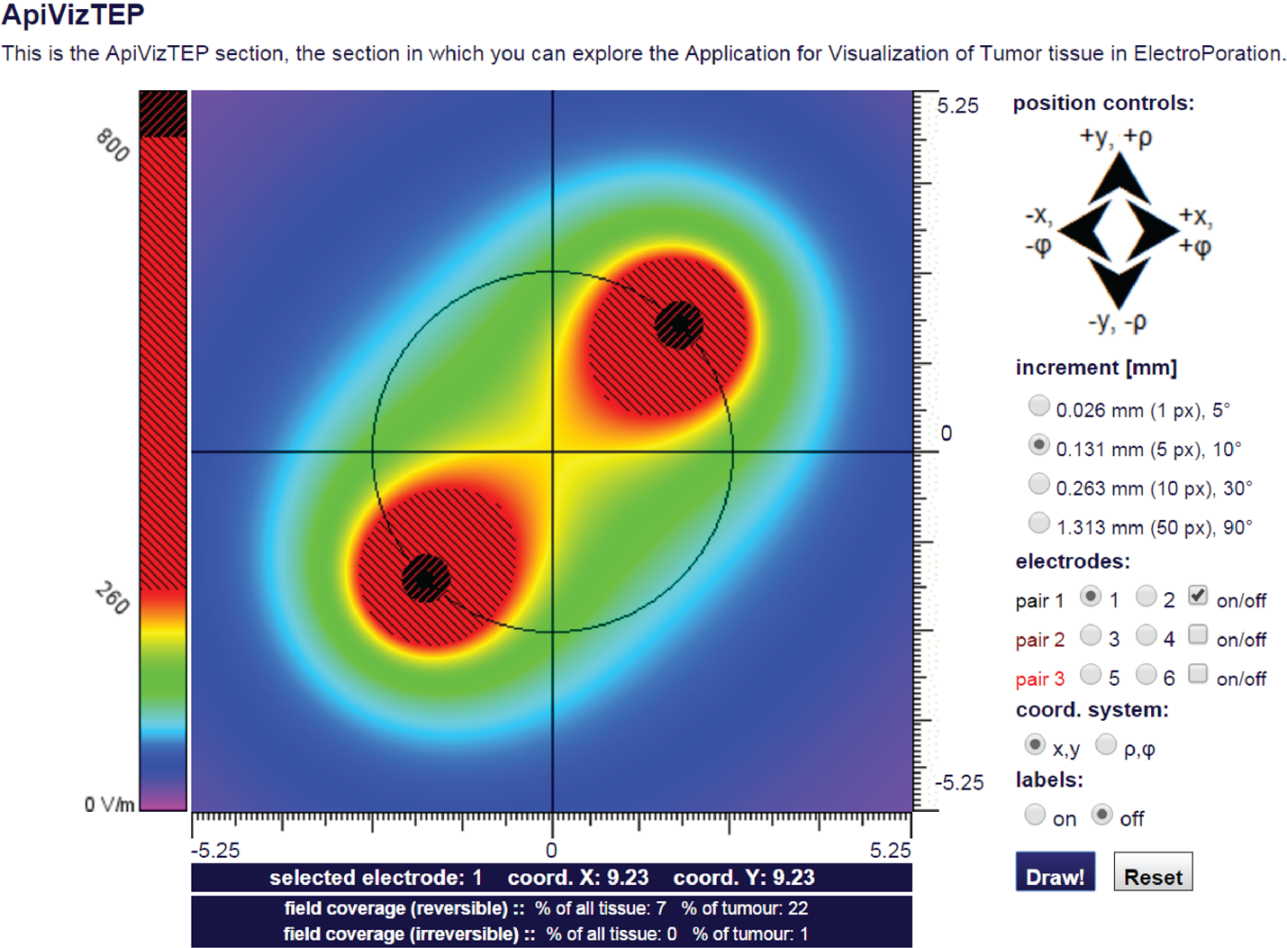
Visualization of analytically calculated electric field distribution within a homogeneous tissue in 2D. Interactive visualization of analytically calculated electric field distribution within homogeneous tumour tissue in 2D (as calculated by the ApiVizTEP interactive application)

Local electric field (Fig. 3c)—this panel provides access to an advanced level of knowledge on electric field distribution in electroporated tissues. The scientific visualization in this panel is represented by 2D and 3D interactive animations (i.e. visualization of electric field distribution) that are based on the knowledge gained from our previous numerical and theoretical studies. An example of interactive scientific visualization of electric field distribution calculated for selected parameters in 2D geometry of a homogeneous tumour tissue (i.e. calculated with the ApiVizTEP interactive application) is given in Fig. 7.

The interactive 3D animations underline the most important findings we demonstrated by numerical calculations in realistic numerical models of a single type tissue (e.g. muscle or tumour tissue) and of more complex or composite tissues (e.g. subcutaneous and cutaneous tumour tissue, muscle with skin, etc.). For example, we explain that the electric field distribution within electroporated tissues is strongly influenced by tissues’ properties such as tissue heterogeneity (i.e. a tissue can be composed of several types of tissues having different conductivities) or tissue anisotropy characterizing for example muscle tissue structure. In addition, the educational content implies that a tissue exhibiting homogeneous electric properties in normal conditions (e.g. a non-electroporated tissue) may become anisotropic due to its exposure to electroporation pulses. We further graphically explain the difference between linear (the models that do not take into account changes in tissue conductivity due to electroporation) and nonlinear electroporation models (the models that take into account the changes in tissue conductivity due to electroporation). Namely, we demonstrated before that the non-linear electroporation models are more realistic and provide more precise prediction of successfully electroporated tissue area which directly affects the accuracy of the electroporation-based treatment outcome [11]. In addition, this panel also emphasizes importance of local electric field calculation and visualization in treatment planning of electrochemotherapy of cutaneous, subcutaneous, and deep-seated tumours. Further goal of this panel is to show the importance of electric field distribution visualization in tissues (such as skin and muscle) in therapeutic applications of electroporation such as gene therapy, DNA vaccination, and trans-dermal drug delivery. Our interactive application thus enables the visualization of electric field distribution in muscle tissue electroporated through the skin layer and in muscle electroporated by direct positioning of the electrodes on or insertion of electrodes into the muscle tissue. This latter case simulates the therapeutic situation where the skin layer is removed prior to applying the treatment. The visualization of electric filed distribution in skin and muscle tissue by means of interactive animations are based on our recent theoretical and experimental study, where we demonstrated that, for the same electric field and thus for the same effect of electroporation in the muscle tissue, a transcutaneous electroporation requires higher voltages to be applied between the plate electrodes as compared to the direct muscle electroporation [10].

**Fig. 8:**
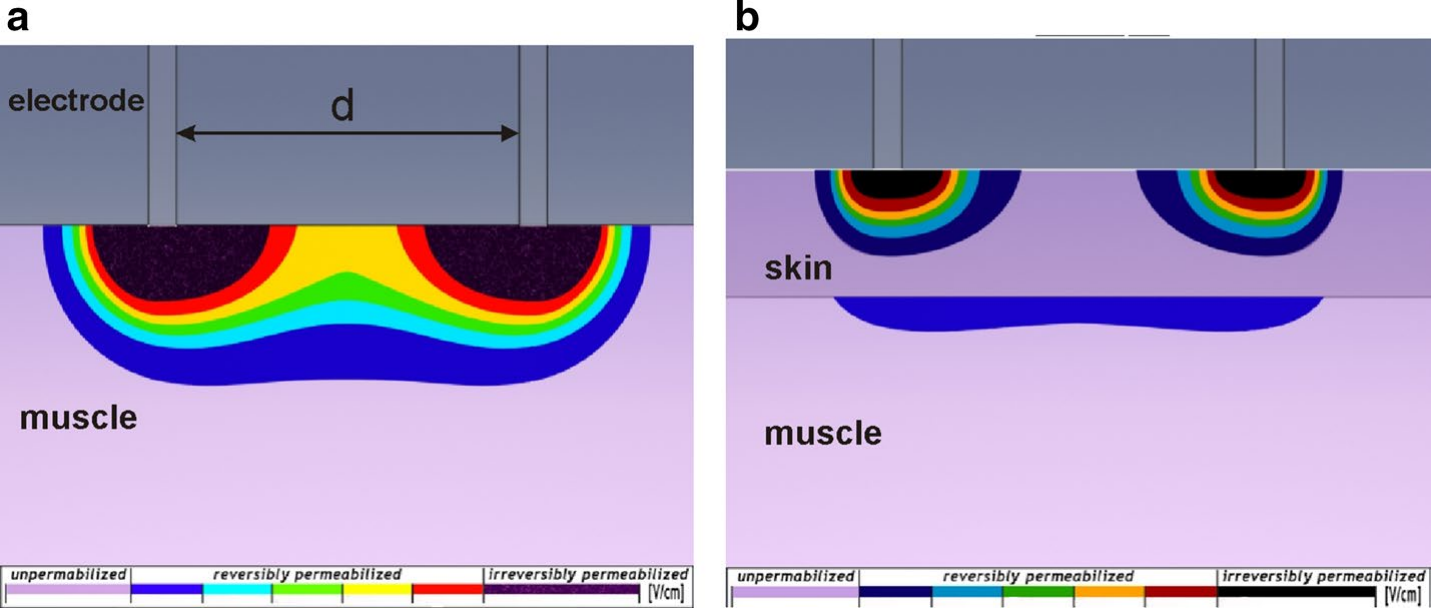
Visualisation of electric field distribution in muscle electroporated directly and transcutaneously. Comparison of electric field distribution in muscle electroporated (**a**) directly and (**b**) transcutaneously. With equal set of plate electrodes and equal voltage applied to the electrodes

In Fig. 8 we show the difference in electric field distribution in muscle electroporated directly and transcutaneously with the same amplitude of voltage applied to the equal set of plate electrodes (i.e. equal dimensions and distance between plates).

Figure 8a shows that a direct exposure of muscle to the electroporation pulses results in higher electric field in a larger area of muscle as compared to electric field distributed in the muscle electroporated through the skin. Figure 8b additionally shows that a high amount of the electric field is retained within the skin layer, while a smaller portion of electric field is distributed within the underlying muscle tissue. This observation has important implications in the field of gene electrotransfer to muscle tissue. Namely, recent preclinical findings in gene electrotransfer to muscle tissue by using transcutaneous electroporation approach resulted in gene expression mainly located in the fatty tissue, while the muscle tissue was not efficiently targeted. The efficacy of gene electrotransfer was than improved considerably by direct electroporation approach i.e. by removing the skin layer prior to the treatment and by electroporating the muscle tissue with electrodes being in direct contact with muscle tissue [24].

### Description of the e-learning practical work delivery

The e-learning practical work we present in this paper was introduced to the students/participants of the International Scientific workshop and postgraduate course EBTT in 2011-2014 academic years. During the four deliveries of the course, there was a total of 47 students/participants coming from different universities, hospitals and research institutions from Europe, USA and Israel, thus representing a mixed audience with heterogeneous experience and knowledge in electrical phenomena involved in electroporation research field. Detailed demographics of participants/students attending the EBTT e-learning practical work in 2011–2014 broken down by professional background, academic degree, and gender are shown in Table 1.

**Table 1:** Demographic characteristic of participants/students attending the EBTT e-learning practical work in 2011–2014 broken down by professional background, academic degree, and gender

	Breakdown
2011–2014: total students 47	
Electrical engineers/natural scientists	13/34
Ph.D. students/researchers with Ph.D.	37/10
Male/female	21/26
Electrical engineers: male/female	8/5
Natural scientists: male/female	18/16
Electrical engineers: Ph.D. students/researchers with Ph.D.	8/5
Natural scientists: Ph.D. students/researchers with Ph.D.	29/5
2011: total students 10	
Electrical engineers/natural scientists	2/8
2012: total students 16	
Electrical engineers/natural scientists	6/10
2013: total students 10	
Electrical engineers/natural scientists	3/7
2014: total students 11	
Electrical engineers/natural scientists	2/9

In order to investigate whether the educational content the e-learning practical work presented to the students with different professional backgrounds enhanced their knowledge acquired via lectures during the EBTT, we compared the learning effect assessed in two experimental groups of students (i.e. total of 13 electrical engineers and 34 natural scientists) undergoing the e-learning practical work in 2011, 2012, 2013, and 2014 academic years. The group of natural scientists was composed of three chemists, seven medical doctors and twenty-four biologists.

The participants were gathered in a computer-equipped classroom providing each participant with a computer equipped with a browser and access to the internet.

The participants were given the instruction to execute the e-learning practical work session according to the following sequence of studying steps: (i) a demographic questionnaire requesting professional background, academic degree and gender information (ii) a pre-test assessing the knowledge achieved via lectures prior to the e-learning session; (iii) studying the e-learning content; (iv) taking the post-test assessing the knowledge after the e-learning course; and (v) concluding with a survey on learning experience and usability of the system. Each of the participants individually completed the evaluation tests and submitted them to the system for further statistical analysis. After completing the usability questionnaire the participants were encouraged to provide their opinion/comments on the practical work and suggestions for its improvement. The e-learning practical work was concluded with the statistical analysis of the data collected with the pedagogical evaluation tests and the usability evaluation test. Finally, the results of the statistical analysis were displayed in the classroom and discussed between the instructors and participants.

The evaluation tests were taken by each of the participants only once and all of the participants were asked to provide their agreement on the use of the results for research purposes.

### Pre and post knowledge assessment and item analysis

The knowledge assessment was conducted with an identical knowledge assessment test administered before (i.e. pre-test) and after (post-test) the e-learning course to all participants/students (i.e. to both experimental groups of students: electrical engineers and natural scientists). The knowledge assessment test was composed of ten questions/items relating to the educational content of the e-learning course. Each correct answer scored one point (otherwise zero point, no negative points were given), giving a maximum test score of ten points. The questions and the corresponding correct answers are given in Additional file 1. In order to investigate whether the e-learning practical work increased the level of students’ knowledge, we calculated and compared the total scores on the pre-test and post-test assessment. As the collected data was non-parametric we used the Wilcoxon signed rank test to determine the statistical difference between the total pre-test score and post-test score achieved within each of the two experimental group and the Wilcoxon rank sum test to determine the statistical difference between the total pre-test score and post-test score achieved between the two experimental groups. A significance level of p < 0.05 was set to all tests.

In order to assess the quality of the items consisting the knowledge assessment test we performed item analysis by calculating difficulty index and discrimination index [25]. Item difficulty expresses the percentage of the participants/students who answered the item correctly and it can range from 0 % (none of the student answered the item correctly) to 100 % (all of the students answered the item correctly). The discrimination index is a parameter that helps to discriminate between the students with a higher and a lower level of knowledge and it can range from −1 to 1. The items with discrimination index above 0.2 are considered to be acceptable. The items with negative discrimination index should be reviewed as they are ambiguous or misleading [25].

### Learning experience and usability evaluation

The learning experience and usability evaluation was performed with a survey consisting of 13 items (Table 3) that asked all the participants/students to rate their perception of learning experience and usability of the e-learning system on a seven point Likert scale (LS) ranging from 1 (disagree—LS1) to 7 (strongly agree—LS7) or to remain neutral by checking neither agree nor disagree (NA) statement, which we considered a neutral evaluation result [26].

## Assessment results and discussion

**Fig. 9:**
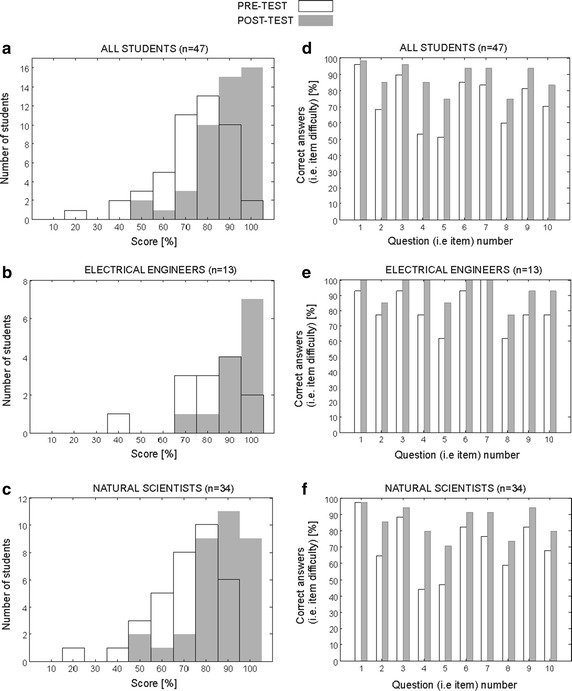
Statistical analysis of the knowledge assessment data collected before (i.e. pre-test) and after (post-test) e-learning practical work. Statistical analysis of the knowledge assessment data collected before (i.e. pre-test) and after (post-test) e-learning course administered to the participants/students of the e-learning practical work. Total score obtained on pre-test versus post-test knowledge assessment **a** all students (i.e. both experimental groups of students: electrical engineers and natural scientists), **b** electrical engineers and **c** natural scientists and percent of correct answers per question/item (i.e. item difficulty): **d** all students (i.e. both experimental groups of students: electrical engineers and natural scientists), **e** electrical engineers, and **f** natural scientists

The results of the statistical analysis of the knowledge assessment data collected before (i.e. pre-test) and after (post-test) e-learning practical work administered to the participants/students of the e-learning practical work of EBTT School in 2011–2014 are summarised in Fig. 9.

The comparison of histograms of total scores obtained on the pre-test versus post-test is given in Fig. 9a–c for all students (i.e. both experimental groups of students: electrical engineers and natural scientists), electrical engineers and natural scientists, respectively.

The percentage of correct answers per pre-test and post-test question/item (i.e. item difficulty) is given in Fig. 9d–f for all students (i.e. both experimental groups of students: electrical engineers and natural scientists), electrical engineers, and natural scientists, respectively.

The results in Fig. 9 show positive learning effects: the total score after the e-learning practical work increased compared to the total score before the e-learning practical work as shown in histograms of total pre- versus post- scores in Fig. 9a (all students—electrical engineers and natural scientists), in Fig. 9b (electrical engineers only), and Fig. 9c (natural scientists only). After the e-learning practical work the total score on the post-test knowledge assessment was equal or exceeded 50 %, and 34 % of participants scored 100 % on the post-test. Statistical analysis showed that the total score on the post-test taken by all students was significantly higher compared to the total score on the pre-test (before taking the e-learning practical work). Similarly, in the total score on the post-test taken by students in both experimental groups was significantly higher.

It is interesting to note that the level of knowledge in the group of electrical engineers was statistically higher compared to the level of knowledge of natural scientists on the pre-examination test, while the level of knowledge obtained after the course was not statistically different. Namely, statistical analysis showed that the electrical engineers significantly outperformed the natural scientists on the pre-test examination while there was no statistically significant difference in their total score on the post-test score examination.

The decrease in item difficulty (i.e. increase in percentage of correct answers) after the e-learning course indicates a positive learning effect of the e-learning practical work as shown in Fig. 9a (all students—electrical engineers and natural scientists) in Fig. 9b (electrical engineers only), and Fig. 9c (natural scientists only). The average percentage of correct answers per question/item ± standard deviation (averaged through all 10 questions/items) and the after-before difference, for all participants and separately for both experimental groups, are listed in Table 2. Results listed in Table 2 indicate that the average difficulty of all items decreased after the e-learning practical work by 14.1 ± 7.0 %. This decrease in item difficulty and the corresponding increase in total score after e-learning practical work is believed to be attributable to the specific knowledge that the participants missed from the live lectures and was delivered via e-learning. The average percentage of correct answer (on pre- and post-tests) was as expected higher for electrical engineers since the educational content of EBTT school is more closely related with existing knowledge of electrical engineers compared to the knowledge of natural scientists. On the second, the post-test take, the average percentage of correct answers per question/item in the group of natural scientists and electrical engineers increased by 14.7 ± 8.2 %, and by 12.3 ± 4.7 %, respectively. This result of positive learning effect we obtained in the group of natural scientists indicates that our blended learning approach could serve as an effective tool in learning on electrical phenomena involved in electroporation-based therapies and treatments also for learning populations without electrical engineering background (e.g. larger classes of undergraduate students in biology, pharmaceutical or medical studies).

**Table 2:** The average increase in percentage of correct answers/item per question/item

	Item difficulty—before [%]	Item difficulty—after [%]	Difference (improvement) [%]
All students/participants (n = 47)	73.6 ± 15.5	87.7 ± 8.5	14.1 ± 7.0
El. engineers (n = 13)	80.8 ± 13.2	93.1 ± 8.5	12.3 ± 4.7
Natural scientists (n = 34)	70.9 ± 17.5	85.6 ± 9.3	14.7 ± 8.2

The average increase in percentage of correct answers/item (averaged through all 10 questions/items), the after-before difference for all participants and separately for all participants, and separately for both experimental groups (i.e. electrical engineers and natural scientists)

**Fig. 10:**
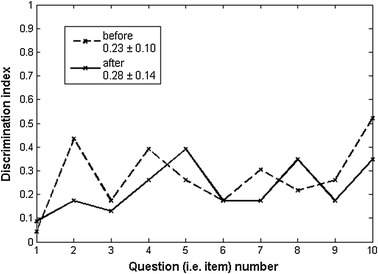
Discrimination index and corresponding mean ± standard deviation values calculated for each item (for all students) before and after e-learning course

The discrimination index for each item calculated for all students before and after taking the exam was positive and the mean value exceeded the acceptable level of 0.2 as shown in Fig. 10. Due to the decrease of item difficulty after the e-learning session the discrimination index as expected also decreased. Namely, the pre-exam measured the level of knowledge achieved with the lectures during EBTT school and the post-test measured the further increase of knowledge level (with the corresponding decrease in item difficulty) achieved with the e-learning practical work. The calculated difficulty index with the corresponding mean ± standard deviation values is given in Fig. 10.

Although a positive learning experience was indicated by our study, there are some aspects of the testing methodology employed that could be improved in future adaptations of the e-learning practical work. Given that the same ten questions were asked before and after the e-learning course, the participants could have been thus clued to look for material for answers to specific questions that they were not sure of or didn’t know. In order to improve on the objectivity of this pre- and post-course tests, we intend to upgrade the tests to include a bank of questions that will ensure the testing is more broadly based. Alternatively, in order to test the same facts, the bank of questions could be complemented by different types of questions designed to test the same material, but asked differently in the pre- and post-test.

### Learning experience and usability evaluation

**Fig. 11:**
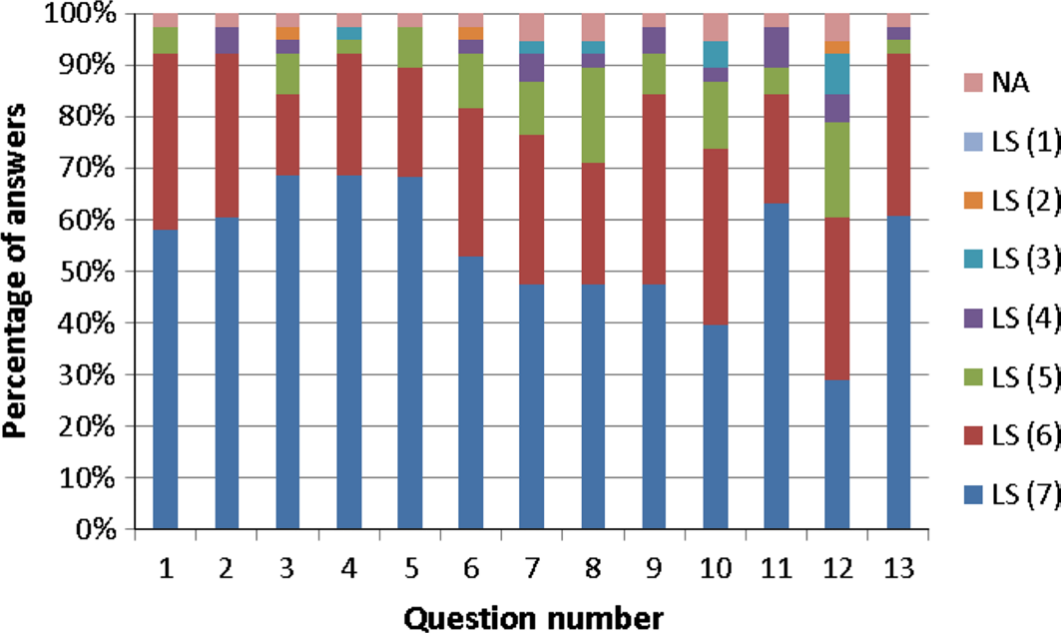
Results of the learning experience and usability evaluation based on seven point Likert scale survey

Statistical analysis of the learning experience and usability evaluation of the data collected via a seven-point Likert scale survey given in Table 3 is summarised in Fig. 11.

**Table 3:** The usability evaluation questionnaire

No.	Question/statement
1	Overall, I am satisfied with how easy it is to use e-learning
2	It was simple to use e-learning
3	I can effectively navigate e-learning
4	I feel comfortable using e-learning
5	It was easy to learn to use e-learning
6	I believe I became more confident using e-learning
7	The information (such as online help, on-screen messages, and other documentation) provided with e-learning is clear
8	It is easy to find the information I needed
9	The information provided for e-learning is easy to understand
10	The information is effective and complete
11	The interface of e-learning is pleasant
12	E-learning covers all the areas I expect it to cover
13	Overall, I am satisfied with e-learning

Questions/statements (13) posed to the students in the usability evaluation questionnaire. Possible answers range on a seven-point Likert-scale, with ‘NA’ option for the neutral opinion

The results of the learning experience and usability evaluation reveal that users find the graphical user interface and the platform effective, easy to understand and to navigate, and they are comfortable using it. However, the users were least in agreement with the statement that e-learning covers all the areas they would expect it to cover. This task of widening the scope of topics covered has become more demanding starting in 2012, when electroporation applications for food and environmental technologies were added to the biomedical field applications, already covered during the past incarnations of the EBTT School. In future we plan to address this issue by further developing the content to better support all lectures given during the course of the School. In addition, we plan to develop educational material covering two areas from the biomedical field, in which the e-learning course presently is particularly lacking in content; the irreversible electroporation, and nanosecond pulse electroporation applications. Furthermore, we plan to perform an eye tracking study and to implement an adaptive e-learning environment that would enable personalisation of the e-learning content based on user’s preferences, feedback, or level of pre-existing knowledge.

In addition, the usability evaluation shows that there is still room for improvement in the design of the e-learning software so as to improve on the participant’s confidence (question 6), clarity of material (q. 7), the ease of finding the information (q. 8), and ease of understanding of the material delivered via this e-learning practical work (q. 9), see Fig. 11. In these aspects, the relatively low percentage of highest score that was attributed may indicate that for students, quick command of the software was perhaps not straightforward enough. Question 10, which had a low response for the highest Likert score for effective and complete information, may be related to the responses to question 12 on the completeness of the material covered, or to those from questions 6–9. In order to address this issue, further work on improving the software will be aimed towards improvements of the user friendliness of the software based on specific comments and suggestions collected from the students during and after future practical work sessions.

## Conclusions

In this paper we present an e-learning practical work we developed to complement delivery of knowledge via lectures and laboratory work, thus providing a blended learning approach on electrical phenomena involved in electroporation-based therapies and treatments. The main objective was to approach users with different professional backgrounds and heterogeneous knowledge (i.e. electrical engineers and natural scientists). The knowledge assessment indicate that our e-learning practical work supported by blended learning approach provides an effective learning tool for populations with different professional backgrounds such as medical doctors, chemists, biologists and electrical engineers.

The important feature of the e-learning application we present in this paper is its modular structure that allows for the continuous upgrade with new content based on new results being published within the scientific literature of electroporation-based therapies and treatments, such as for example electrochemotherapy and electroporation-based gene therapy and DNA vaccination, soft tissue ablation by irreversible electroporation, nanopulse electroporation, electroporation-based food processing, and environmental technologies.

## Additional file

10.1186/s12938-016-0152-7 Knowledge assessment test with the corresponding correct answers. The knowledge assessment test was composed of ten questions related to the educational content of the e-learning practical work. The questions and the corresponding correct answers to the questions.
