# Supplementary material for: Education on electrical phenomena involved in electroporation-based therapies and treatments: a blended learning approach
Source: Biomed Eng Online. 2016 Apr 7;15:36. doi: 10.1186/s12938-016-0152-7 (PMC4823865; doi:10.1186/s12938-016-0152-7)
Supplement: Supplementary file 1 — 10.1186/s12938-016-0152-7 Knowledge assessment test with the corresponding correct answers. The knowledge assessment test was composed of ten questions related to the educational content of the e-learning practical work. The questions and the corresponding correct answers to the questions. [file 12938_2016_152_MOESM1_ESM.pdf]

Pedagogical efficiency questionnaire with the correct answers

1. The requirement for effective electrochemotherapy in terms of local electric field distribution E is the following:

- (●) entire tumor tissue has to be exposed to E above reversible threshold E_{rev}
- ( ) entire tumor tissue has to be exposed to E below reversible threshold E_{rev}

2. Which parameters have to be optimized in order to obtain the appropriate local electric field inside the tumor and its surrounding healthy tissues?

- [x] electrode geometry
- [x] position of electrodes
- [x] applied voltage
- [ ] the dose of bleomycin

(multiple answers are possible)

3. A spherical cutaneous tumor (2r = 4 mm) shown in Figure 1 has to be treated with ECT using 2 parallel plate electrodes. Currently applied voltage (U = 300 V), distance between electrodes (8 mm) and electrode dimensions (4 mm wide) are not appropriate parameters. In order to obtain higher local electric field distribution inside the tumor the distance (d) between electrodes has to be:

- (●) decreased
- ( ) increased

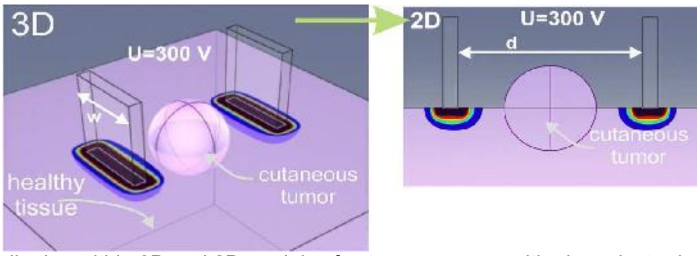

Figure 1: Electric field distribution within 3D and 2D models of cutaneous tumor with plate electrodes, where distance between electrodes d = 8 mm, electrode width w = 4 mm and the applied voltage U = 300 V

4. The local electric field distribution E within the target tissue is shown in Figure 2, when a pair of needle electrodes (diameter 2r = 0.45 mm) is inserted. If you replace this pair with a pair of needle electrodes of a smaller diameter 2r < 0.45mm, the E within the target tissue will

- ( ) increase
- ( ) remain unchanged
- (●) decrease

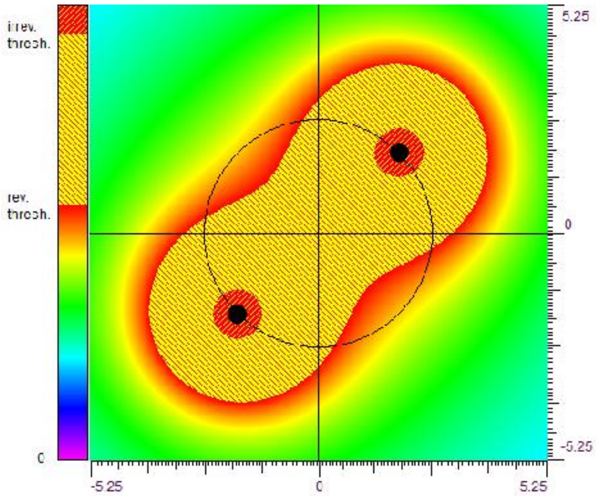

Figure 2: Two-needle situation modeled in ApiVizTEP

5. In order to treat the tumor in Figure 1 you can use a pair of plate electrodes with w = 4 mm, w = 7mm or w =10 mm. When the distance (d) between the electrodes and the applied voltage (U) are fixed at d = 5 mm and U = 900 V the entire tumor tissue (see Figure 1) is exposed to $E \geq E_{rev}$. Which pair of electrodes would you use in order to prevent damages to the healthy tissue?

- (●) w = 4 mm
- ( ) w = 7 mm
- ( ) w = 10 mm

6. If the plate electrodes are too short with respect to the tumor tissue (see Figure 3) or the applied voltage on the electrodes is too low the following tumor response can be expected:

- ( ) tumor cells regrow in the central region of the tumor (Region 1)
- ( ) tumor cells regrow in close proximity of the electrodes (Region 2)
- (●) tumor cells regrow in regions which are far from the electrodes (Region 3)

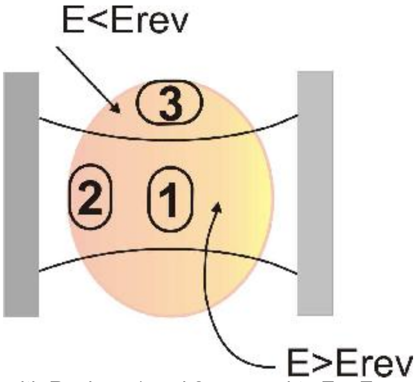

Figure 3: Illustration of a tumor with Regions 1 and 2 exposed to $E > E_{rev}$ and Region 3 exposed to $E < E_{rev}$

7.The entire region of the target tissue shown in Fig. 4 is not exposed to the local electric field above E_{rev} (E_{rev} > 260 V/cm). Which solution would be more adequate in order to cover entire area of target tissue with $E > E_{rev}$ with minimal exposure of the tissue to $E > E_{irrev}$?

- ( ) increase the applied voltage to the inserted electrodes (pair 1)
- (●) insert an additional pair of electrodes (pair 2) and keep the same voltage (200 V)

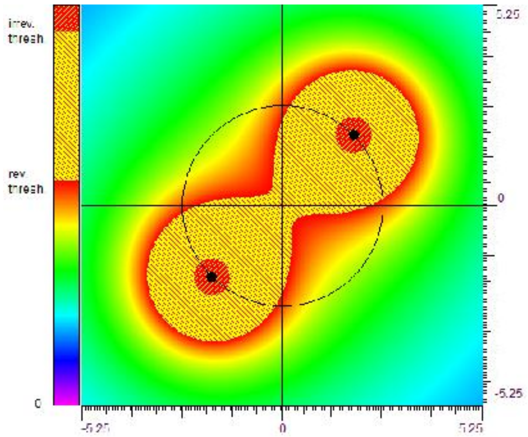

Figure 4: Two-needle situation modeled in ApiVizTEP

8. Due to electropermeabilisation of the skin layer, local electric field within underlying tissues:

- (•) increases
- ( ) decreases

9. Which of the following needle electrode configurations will result in higher local electric field inside the subcutaneous tumors shown in Figure 5 (if the applied voltage U is the same in both cases)?

- ( ) two needle electrodes (one-pair)
- (•) a parallel array of six needle electrodes (three-pairs)

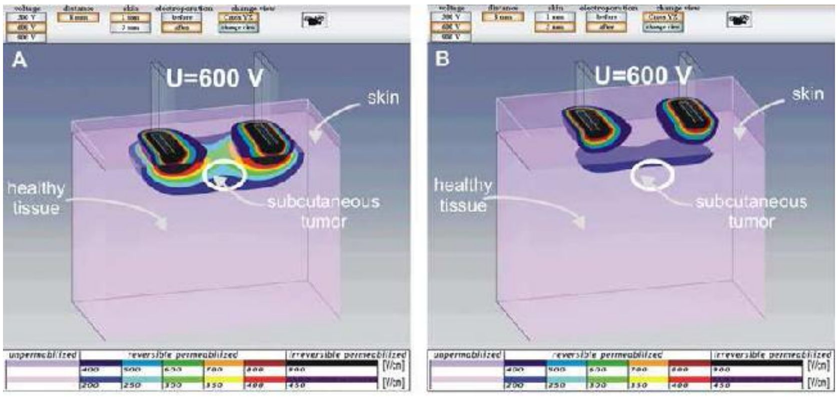

Figure 5: Electric field distribution within a model of subcutaneous tumors seeded: A. below a 1 mm thick skin layer; B. below a 3 mm thick skin layer

10. See the electric field distribution within the target subcutaneous tumor seeded below a 3 mm thick skin layer in Figure 5B. If you had only the following two possibilities to increase the local electric field inside the tumor, which one would you choose in order to reversibly electroporate the tumor and protect the surrounding healthy tissue:

- ( ) increase the applied voltage to U = 900 V
- (•) insert a parallel array of six needle electrodes (three pairs) and apply U = 300 V
